# A Novel DNA Vaccine Against SARS-CoV-2 Encoding a Chimeric Protein of Its Receptor-Binding Domain (RBD) Fused to the Amino-Terminal Region of Hepatitis B Virus preS1 With a W4P Mutation

**DOI:** 10.3389/fimmu.2021.637654

**Published:** 2021-02-26

**Authors:** Hyein Jeong, Yu-Min Choi, Hyejun Seo, Bum-Joon Kim

**Affiliations:** Department of Biomedical Sciences, College of Medicine, Microbiology and Immunology and Liver Research Institute, Seoul National University, Seoul, South Korea

**Keywords:** SARS-CoV-2, COVID-19, DNA vaccine, HBV preS1, Receptor-binding domain (RBD), W4P-RBD

## Abstract

A coronavirus SARS-CoV-2, which has caused the pandemic viral pneumonia disease COVID-19, significantly threatens global public health, highlighting the need to develop effective and safe vaccines against its infection. In this study, we developed a novel DNA vaccine candidate against SARS-CoV-2 by expressing a chimeric protein of its receptor-binding domain (RBD) fused to a 33-bp sequence (11 aa) from the hepatitis B virus (HBV) preS1 region with a W4P mutation (W4P-RBD) at the N-terminal region and evaluated its immunogenicity. *In vitro* transfection experiments in multiple cell lines demonstrated that W4P-RBD vs. wild-type RBD protein (W-RBD) led to enhanced production of IL-6 and TNFα at the transcription and translation levels, suggesting the adjuvant potential of N-terminal HBV preS1 sequences for DNA vaccines against SARS-CoV-2. W4P-RBD also led to enhanced production of IgG and IgA, which can neutralize and block SARS-CoV-2 infection in both blood sera and bronchoalveolar lavage (BAL) fluid from the lung in vaccinated mice. Additionally, W4P-RBD led to an enhanced T-cell-mediated cellular immune response under S1 protein stimulation. In summary, W4P-RBD led to robust humoral and cell-mediated immune responses against SARS-CoV-2 in vaccinated mice, highlighting its feasibility as a novel DNA vaccine to protect against SARS-CoV-2 infection.

## Introduction

The coronavirus disease 2019 or COVID-19 pandemic is caused by severe acute respiratory syndrome coronavirus 2 (SARS-CoV-2) (1–5), highlighting the need to develop effective and safe vaccines against its infection. Similar to SARS-CoV, SARS-CoV-2 recognizes angiotensin-converting enzyme 2 (ACE2) as a receptor for host cell entry (6, 7). The SARS-CoV-2 spike (S) protein consists of S1, including the receptor-binding domain (RBD) and S2 subunits (8). The RBD in both SARS-CoV and SARS-CoV-2 infections is required for ACE2 receptor docking (6, 9–11), and most of the potent neutralizing monoclonal antibodies have been produced against the SARS-CoV-2 RBD (12–14), indicating that the RBD is an attractive vaccine target against SARS-CoV-2 infections. To date, several RBD-targeting vaccines against SARS-CoV-2 include mRNA- (15, 16) or protein-based subunit vaccines (17, 18). However, despite the advantages of the RBD as a vaccine target, DNA-based vaccines targeting RBD alone have not been developed, possibly due to their relatively low immunogenicity by the relatively small length of antigen (19). Therefore, to develop RBD-based DNA vaccines, strategies to promote immunogenicity, including the use of appropriate adjuvants or addition of exogenous sequences capable of potentiating immune responses, should be combined (20–25).

Previously, we reported that the hepatitis B virus preS1 variant with the W4P mutation, in which tryptophan is changed to proline at the fourth codon from the start of preS1, could contribute to hepatocellular carcinoma (HCC) progression in chronic hepatitis B male patients by enhanced IL-6 production (26–28). This prompted us to hypothesize that the preS1 sequence of the W4P HBV variant could act as a booster sequence for DNA vaccines or several types of virus-based vaccines carrying DNAs (adenovirus- or poxvirus-based vaccines).

**Figure 1:**
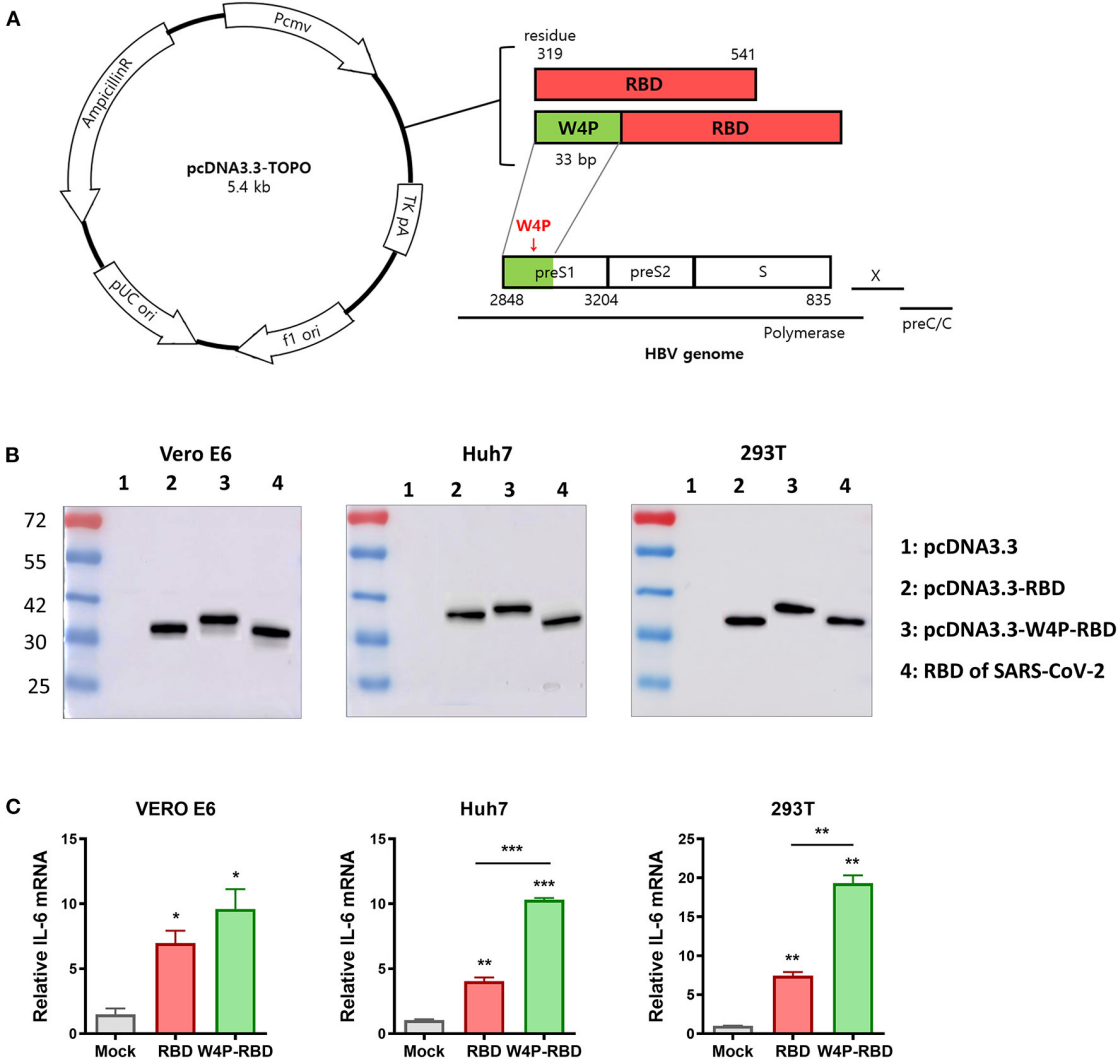
Construction of the HBV W4P preS1-fused pcDNA3.3-RBD plasmid (W4P-RBD) as a candidate for SARS-CoV-2. **(A)** Design of pcDNA3.3-RBD and pcDNA3.3-W4P-RBD. The W4P region comprises 33 bp from the first site of the preS1 region of the HBV genome and encodes 11 amino acids. **(B)** The protein expression of SARS-CoV-2 RBD and W4P-conjugated RBD was detected by the Western blot assay. pcDNA3.3-RBD, pcDNA3.3-W4P-RBD, and empty pcDNA3.3 were transfected into Vero E6, Huh7, and 293T cells, and cell lysates were collected 48 h post transfection to detect protein expression. **(C)** The mRNA expression levels of IL-6 and in pcDNA3.3-transfected cells were detected by qRT-PCR. Significance differences (*P < 0.05, **P < 0.01, ***P < 0.001) among the different groups are shown in the related figures, and the data are presented as the means ± s.e.m. of three independent experiments.

Therefore, in this study, to strengthen the immunogenicity of conventional RBD-based DNA vaccines (designated W-RBD), we sought to develop a novel RBD-based DNA vaccine against SARS-CoV-2 encoding a chimeric protein of the receptor-binding domain (RBD) fused to a 33-bp (11 aa) preS1 sequence of the HBV W4P variant at the N-terminal region (designated W4P-RBD) (Figure 1A). Its potential as a DNA vaccine against SARS-CoV-2 was evaluated using *in vitro* and *in vivo* experiments. First, W4P-RBD can leads to enhanced cytokine production in several transfected cell lines, suggesting a role as an adjuvant of the N-terminus-added HBV sequence in RBD-based DNA vaccines. Second, W4P-RBD also leads to an enhanced cell-mediated immune responses, higher functional IgG and IgA production, which can neutralize and block SARS-CoV-2 infection in vaccinated mice. Furthermore, antibodies in sera or BAL fluid from W4P-RBD-vaccinated mice show enhanced cell entry inhibition of live virus or pseudotyped virus into ACE2-producing cells Huh-7, Calu-3, and Vero-E6 at all dilutions, suggesting W4P-RBD does not promote ADE.

## Results

### Design and Construction of the HBV W4P preS1-Fused pcDNA3.3-RBD Plasmid (W4P-RBD) as a DNA Vaccine Candidate for SARS-CoV-2

Our previous studies have demonstrated that a preS1 W4P substitution, in which tryptophan is changed to proline at the fourth codon of the HBV preS1 region, is related to HCC in chronic male patients via enhanced IL-6-mediated inflammation (27), suggesting the adjuvant potential of the W4P preS1 region for DNA vaccines. Therefore, in this study, to maximize the immunogenic efficacy of DNA vaccines, we constructed an HBV W4P preS1-fused pcDNA3.3-RBD plasmid (designated W4P-RBD, 235 aa) expressing a chimeric protein, in which the first 33 bp encoding 11 amino acids from the start codon of HBV W4P preS1 as a vaccine adjuvant was fused to the N-terminal region at RBD (residues 319–541 of the spike protein) of SARS-CoV-2 (Figure 1A). Its DNA vaccine efficacy was compared with that of the pcDNA3.3-RBD plasmid (designated W-RBD, 224 aa) adding only the start codon (methionine) to the N-terminus of the RBD. We measured the expression of the encoded SARS-CoV-2 RBD transgene at the protein level in Vero E6, Huh7, and 293T cells transfected with the constructed plasmids W-RBD and W4P-RBD via Western blot analysis using an antibody against SARS-CoV-2 RBD in cell lysates. Western blots of the lysates of transfected cells demonstrated that both W-RBD and W4P-RBD produced the expected RBD protein expression in all transfected cells at 48 h post transfection (Figure 1B). Although W4P-RBD revealed bands approximating the predicted RBD protein molecular weight (27–30 kDa, similar to that of the RBD protein control), W4P-RBD revealed bands slightly larger than that of W4P-RBD or the control because of the addition of the preS1 W4P region of 11 aa (length 235 aa). Our qRT-PCR data showed that the mRNA expression levels of inflammatory cytokines IL-6 and TNF-α, capable of potentiating vaccine efficacy, were significantly elevated by W4P-RBD in all the transfected cell lines compared with the W-RBD- and mock-transfected cells (Figure 1C, Supplementary Figure 1A). Consistently, our ELISA data also showed that TNF-α production from W4P-RBD-transfected cells was significantly enhanced in all the transfected cell lines (Supplementary Figures 1B,C). These results suggest the vaccine adjuvant effect of the W4P preS1 region in W4P-RBD as a DNA vaccine candidate for SARS-CoV-2 infection.

### W4P-RBD Leads to an Enhanced Humoral Immune Response Against SARS-CoV-2 Infection in the Sera of Vaccinated Mice

**Figure 2:**
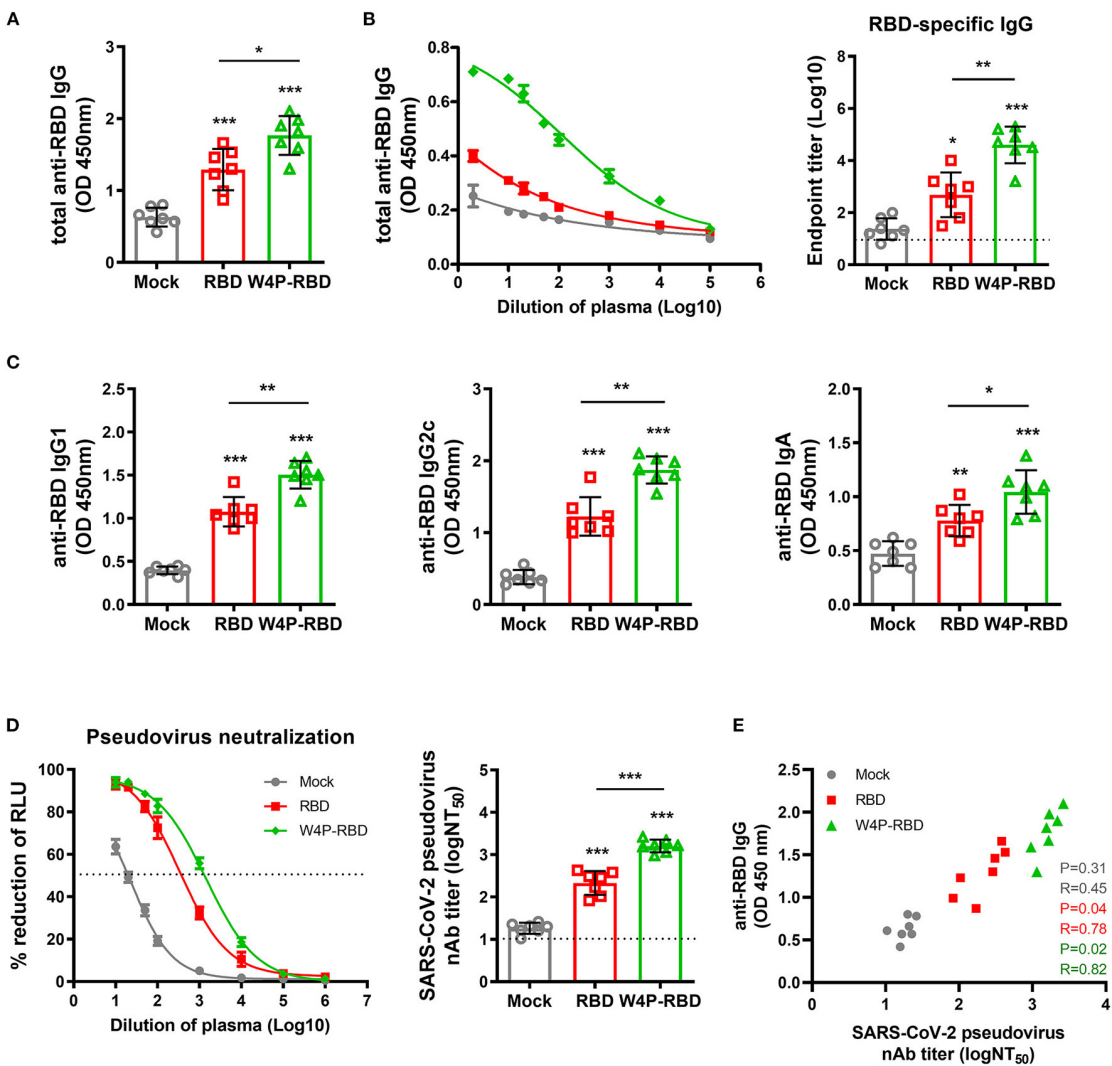
W4P-RBD elicits RBD-specific antibody responses in serum and induces potent neutralizing activity against pseudotyped SARS-CoV-2. C57BL/6 mice were immunized with W-RBD, W4P-RBD (50 μ*g*/mouse), or empty pcDNA3.3 (Mock) three times at 1-week intervals. **(A,C)** Antibody responses in serum specific to SARS-CoV-2 RBD proteins were detected by ELISA. **(B)** Serum at 5 weeks after the last immunization was assessed using different dilution factors for IgG against the SARS-CoV-2 RBD protein using ELISA. **(D)** The 50% neutralizing antibody titer (NT_50_) was calculated using the SARS-CoV-2 pseudovirus neutralization assay in Calu-3 cells. **(E)** Correlation between SARS-CoV-2 RBD-specific IgG and pseudotyped SARS-CoV-2 neutralization titers for immunized mice. Significance differences (*P < 0.05, **P < 0.01, ***P < 0.001) among the different groups are shown in the related figures, and the data are presented as the means ± s.e.m. of mice (*n* = 7). Pearson's correlations were calculated to define correlations.

Next, we evaluated humoral immune responses and neutralizing antibodies induced by W-RBD and W4P-RBD in vaccinated mice. C57BL/6 mice were i.m. injected with plasmid DNAs, W-RBD and W4P-RBD, or mock with a schedule of three times at 1-week intervals. Five weeks after the last injection, the sera were collected to detect the total IgG, IgG subtypes (IgG1 and IgG2c), IgA, and neutralizing antibodies. Our ELISA data showed that W4P-RBD led to significantly elevated production of total IgG, IgG1, IgG2c, and IgA against SARS-CoV-2-RBD or S1 proteins in immunized mouse sera compared with that in the mock or W-RBD group (Figures 2A,C, Supplementary Figure 2A). The total IgG titer against the SARS-CoV-2 RBD protein in the mouse group vaccinated with W4P-RBD was also always higher in the sera of different dilutions than that in the mock or W-RBD group (Figure 2B,) left panel. We also measured the serum IgG binding endpoint titers (EPTs) in mice immunized with plasmid DNAs against recombinant SARS-CoV-2 RBD. Significantly enhanced EPTs were also observed in the sera of mice vaccinated with W4P-RBD (Figure 2B, right panel).

Next, we assessed the neutralizing activities in mouse sera using a reporter lentivirus-based pseudovirus. Neutralization titers were detected by a reduction in the relative luciferase units (RLU) compared with controls. Sera collected 5 weeks after the last immunization were used for the neutralizing assay. The pseudovirus was incubated with serial dilutions of mouse sera, and the sera-virus mixture was added to Huh-7, Calu-3, and Vero-E6 cells for 48 h. Pseudovirus neutralization assays showed that both W-RBD and W4P-RBD elicited neutralizing antibodies against SARS-CoV-2 pseudovirus entry into Huh-7, Calu-3 and Vero-E6 cells; in particular, W4P-RBD elicited significantly higher titers of neutralizing antibodies than W-RBD (Figure 2D, Supplementary Figure 3A). Consistently, BAL fluid from immunized mice was collected and used to detect IgG, IgA, and neutralizing antibodies. W4P-RBD led to enhanced induction of RBD- and S1-specific IgG and IgA and pseudovirus neutralizing antibody titers compared with W-RBD (Supplementary Figures 2B, 3B, 4A–C). These data suggest that W4P-RBD vaccination induces strong RBD-specific antibody responses and potent neutralizing antibodies against pseudotyped SARS-CoV-2 in vaccinated mouse sera.

### W4P-RBD Exerts Neutralizing Activity Against Live SARS-CoV-2 in the Sera of Vaccinated Mice

**Figure 3:**
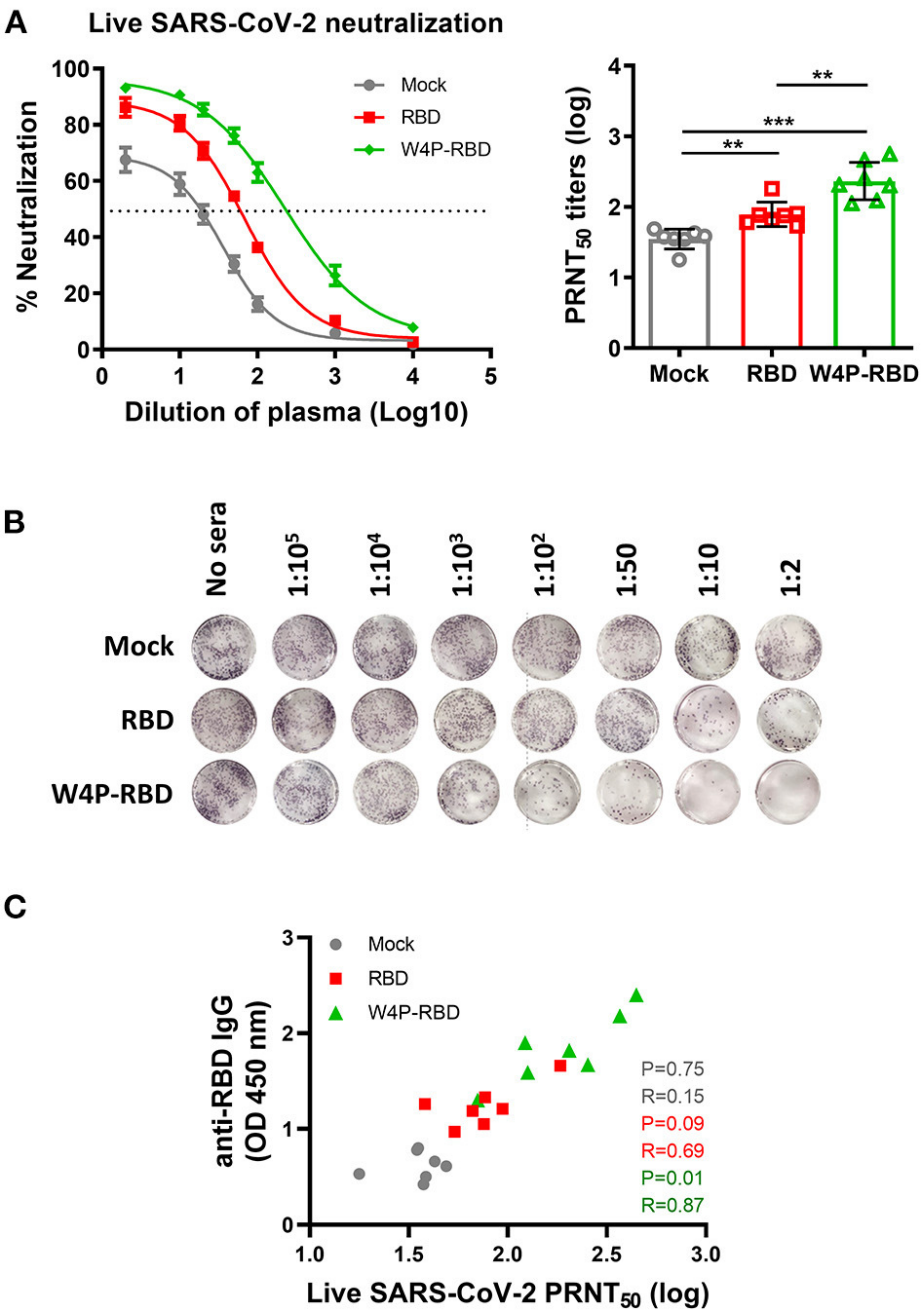
W4P-RBD exerts potent neutralizing activity against live SARS-CoV-2. C57BL/6 mice were immunized with W-RBD, W4P-RBD (50 μ*g*/mouse), or empty pcDNA3.3 (Mock) three times at 1-week intervals. Serum from the immunized mice was diluted and incubated with live SARS-CoV-2 for neutralization assays. **(A)** A 50% plaque reduction neutralizing antibody (PRNT_50_) titer against live SARS-CoV-2 was calculated against SARS-CoV-2 infection in Vero E6 cells. **(B)** Reduction in plaque formation in Vero E6 cells infected with SARS-CoV-2. **(C)** Correlation between SARS-CoV-2 RBD-specific IgG and SARS-CoV-2 neutralization titers in immunized mice. Significance differences (**P < 0.01, ***P < 0.001) among the different groups are shown in the related figures, and the data are presented as the means ± s.e.m. of mice (*n* = 7). Pearson's correlations were calculated to define correlations.

Additionally, neutralizing titers were measured against a live SARS-CoV-2 virus strain using the plaque reduction neutralization test (PRNT) assay. Similar to the pseudotyped virus, ~150 pfu of live SARS-CoV-2 was incubated with serially diluted mouse sera, and the sera-virus mixture was infected into Vero E6. Consistently, higher PRNT_50_ titers were found in the W4P-RBD group than in both the W-RBD and mock groups (Figure 3A, Supplementary Figure 5A), suggesting that W4P-RBD can potently neutralize live SARS-CoV-2 infection. No antibody-dependent enhancement (ADE) was observed in the PRNT assay (Supplementary Figure 5C). The neutralizing activity of the serum group was consistently observed in the case of BAL fluid (Supplementary Figures 4D, 5B). Because SARS-CoV-2 neutralizing activity relies on antibody responses, we assessed whether PRNT_50_ titers were associated with anti-RBD IgG production. A significant correlation between anti-RBD IgG in sera and PRNT_50_ titers was observed in the W4P-RBD group but not in the W-RBD and mock groups (Figure 3C). These results revealed that the neutralizing activity of sera against live SARS-CoV-2 was highest in the W4P-RBD group and had a positive correlation with increased antibody responses only in the W4P-RBD group.

### The Serum From W4P-RBD-Vaccinated Mice Exerts Enhanced Antiviral Effects

**Figure 4:**
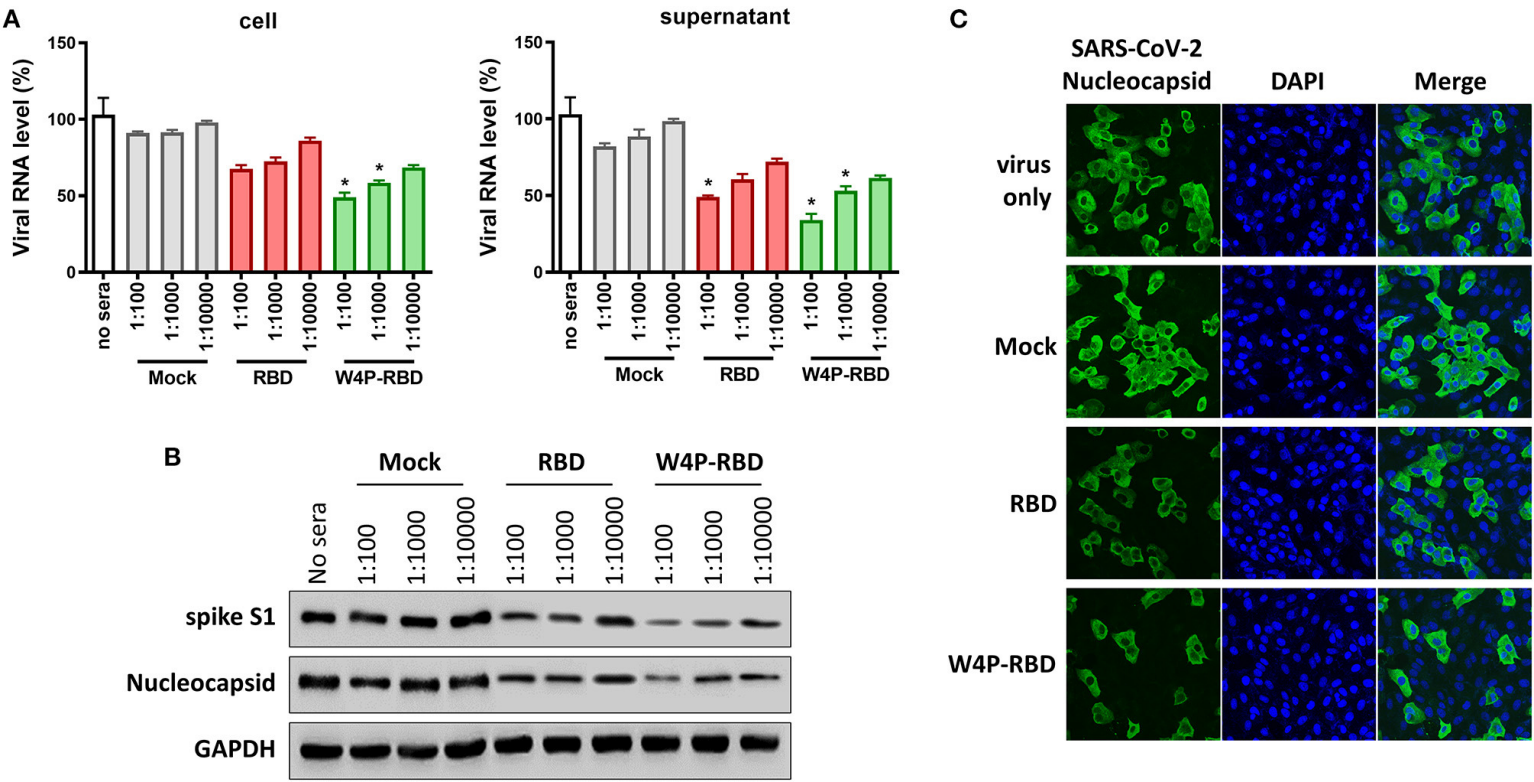
The W4P-RBD vaccine exerts potent neutralizing activity against live SARS-CoV-2 and inhibits viral infection and replication of SARS-CoV-2. C57BL/6 mice were immunized with W-RBD, W4P-RBD (50 μ*g*/mouse), or empty pcDNA3.3 (Mock) three times at 1-week intervals. Serum from the immunized mice was diluted and incubated with live SARS-CoV-2 for neutralization assays. **(A)** The neutralization efficacy of serum from immunized mice against live SARS-CoV-2 RNA copy number in Vero E6 cells was determined by qRT-PCR. **(B)** The neutralization efficacy of the diluted serum against live SARS-CoV-2 in Vero E6 cells was determined by Western blotting. **(C)** Pooled serum (diluted 1:500) from each mouse group was tested in the neutralization assay against live SARS-CoV-2. Representative images (40-fold magnification) show live SARS-CoV-2-infected cells after neutralization in each group. Significance differences (*P < 0.05) among the different groups are shown in the related figures, and the RNA data are presented as the means ± s.e.m. of mice (*n* = 7).

Next, we further checked the antiviral potential of sera within W4P-RBD-vaccinated mice. To this end, the antiviral effect of sera from vaccinated mice in infected Vero E6 cells was evaluated via qPCR, Western blot analysis and IFA. First, the mRNA levels of RNA-dependent RNA polymerase (RdRp) of live SARS-CoV-2 in infected Vero E6 cell lysates or culture supernatants were the most potently inhibited in the W4P-RBD group using the sera at different dilution folds (1:100, 1:1,000, and 1:10000) in a dose-dependent manner (Figure 4A). Second, Western blots of the lysates of the infected Vero E6 cells using two different Abs of SARS-CoV-2 S1 and NP also proved that W4P-RBD elicited the most potent antiviral effect using the sera at different dilution folds (1:100, 1:1,000, and 1:10,000) (Figure 4B). Finally, an indirect immunofluorescence assay (IFA) using a polyclonal antibody against SARS-CoV-2 NP proteins revealed an enhanced neutralization effect of W4P-RBD (Figure 4C). Taken together, our data demonstrated that sera from W4P-RBD-vaccinated mice also led to enhanced antiviral effects in infected cells.

### W4P-RBD Leads to Enhanced Cellular Immune Responses Specific to SARS-CoV-2 S1 in Vaccinated Mice

**Figure 5:**
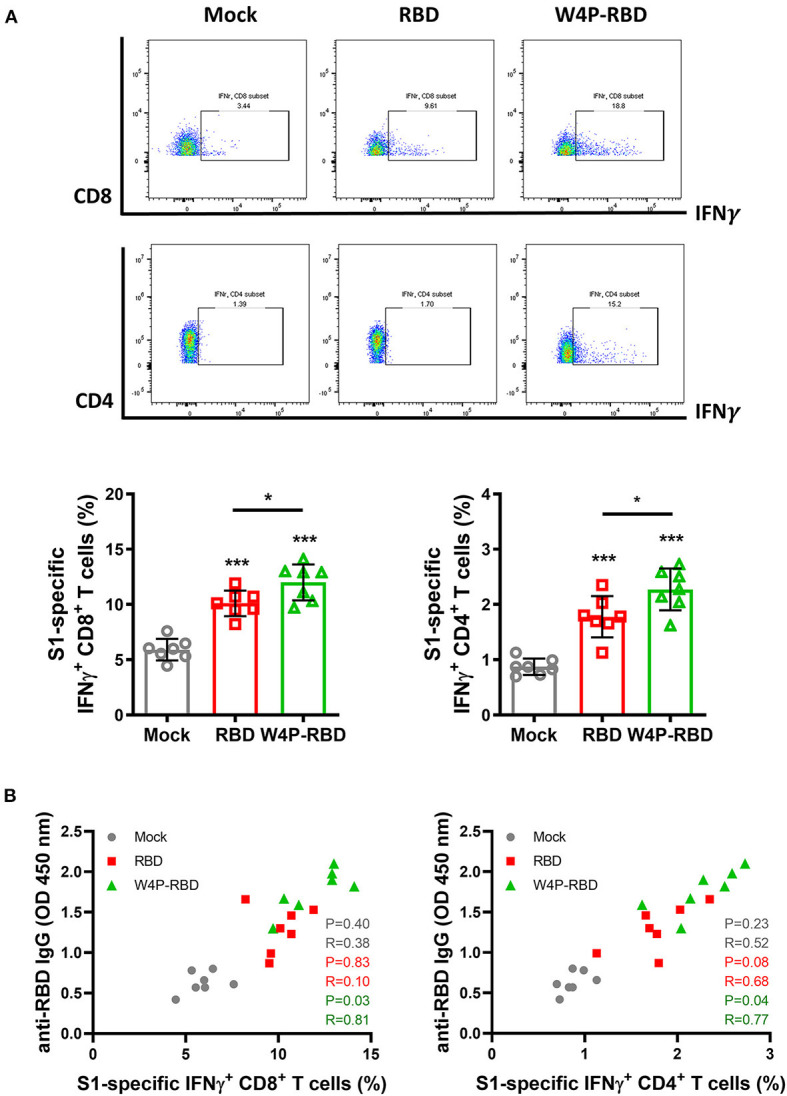
W4P-RBD potentiates functional T cells specific to SARS-CoV-2 S1 proteins. C57BL/6 mice were intramuscularly injected with W-RBD, W4P-RBD (50 μ*g*/mouse), or mock, and the spleens were collected 5 weeks post-vaccination for analysis by flow cytometry. **(A,B)** Splenocytes were incubated with SARS-CoV-2 S1 protein (5 μ*g*/*ml*) for 24 h and stained to detect IFNγ-producing CD8^+^ T cells and CD4^+^ T cells. **(C)** Correlation between RBD-specific IgG in serum and the S1-specific T-cell population in splenocytes. Significance differences (*P < 0.05, ***P < 0.001) among the different groups are shown in the related figures, and the data are presented as the means ± s.e.m. of mice (*n* = 7). Pearson's correlations were calculated to define correlations.

Next, we also characterized the cellular response and induction of systemic cytokines in response to vaccination with W4P-RBD. Splenocytes were harvested from mice at 5 weeks post immunization with W-RBD, W4P-RBD, or mock, and flow cytometry was applied to splenocytes subjected to inoculation with 5 μ*g*/*ml* of SARS-CoV-2 S1 proteins for 24 h. The frequencies of IFNγ- and TNFα-releasing cytotoxic CD8^+^ T cells were the most strongly increased in the W4P-RBD group compared with that in the W-RBD or mock group, suggesting that W4P-RBD led to increased activated CTLs in vaccinated mice. As shown in cytotoxic CD8^+^ T cells, IFNγ- and TNFα-producing CD4^+^ T cells were also the most strongly induced in the W4P-RBD group, suggesting that W4P-RBD also led to increased activated CD4+ helper T cells in vaccinated mice (Figure 5A, Supplementary Figures 6A,B). Because the efficacy of antibody responses relies on T-cell help, we assessed whether SARS-CoV-2 antibody titers were associated with cell-mediated immune responses. Because S1-specific IFNγ-releasing CD4^+^ and CD8^+^ T cells were significantly increased in W4P-RBD compared with that in W-RBD, we assessed whether IFNγ-releasing CD4^+^ and CD8^+^ T cells were associated with antibody titers to the SARS-CoV-2 RBD. No correlation was observed between SARS-CoV-2 antibody titers and cell-mediated immune responses in the W-RBD and mock groups (Figure 5B). Anti-RBD antibody titers developed more strongly in the W4P-RBD group because IFNγ-releasing CD4^+^ and CD8^+^ T cell responses were strongly induced (Figure 5B). Taken together, our data demonstrated that W4P-RBD led to the highest induction of IFNγ-releasing T cells in splenocytes when stimulated with SARS-CoV-2 S1 antigen. Additionally, the cell-mediated immune response induced by W4P-RBD is significantly correlated with IgG antibody responses against SARS-CoV-2 RBD.

### W4P-RBD Induces Proinflammatory Cytokine Production Against SARS-CoV-2 S1 Stimulation in Splenocytes in Vaccinated Mice

**Figure 6:**
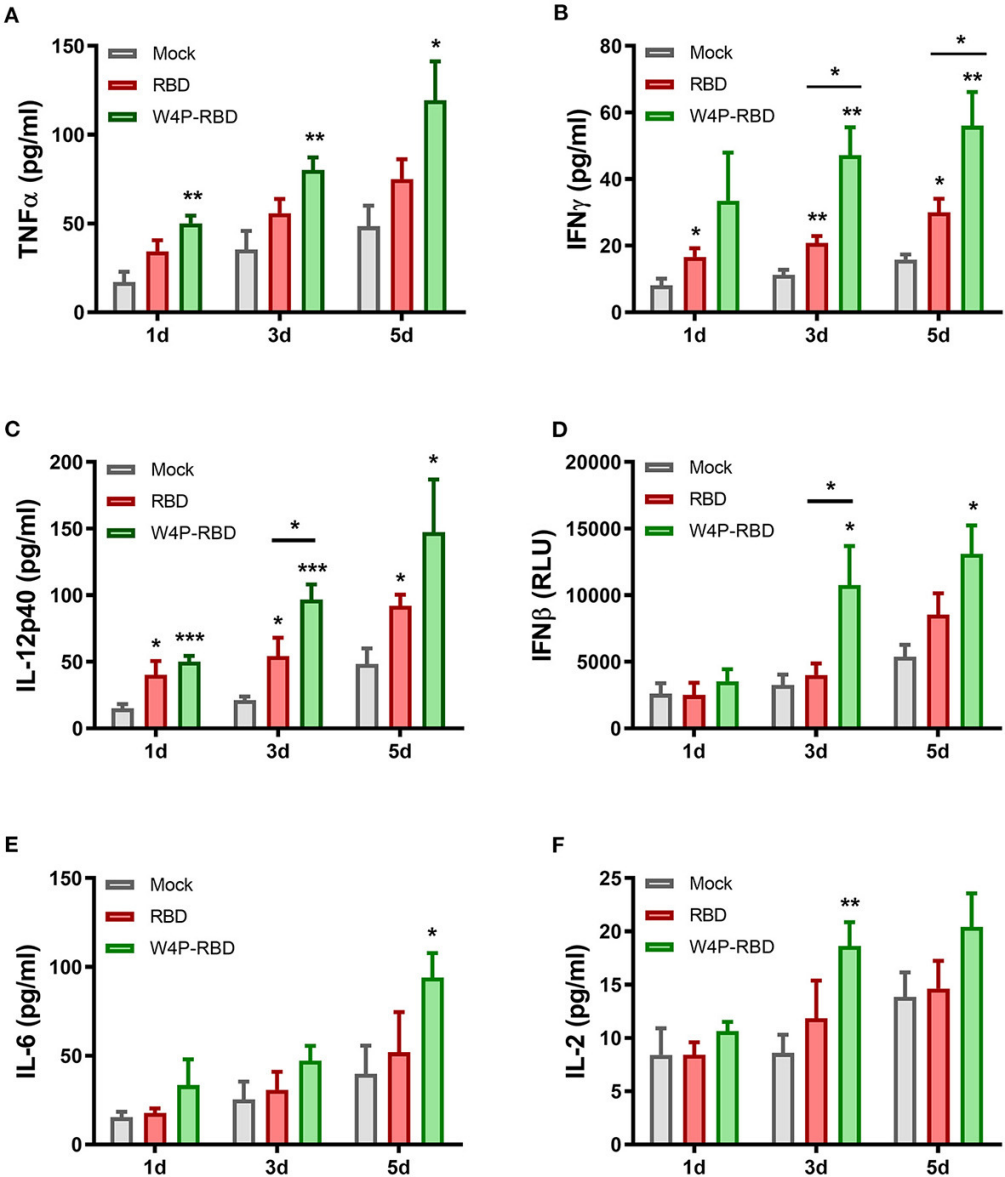
W4P-RBD induces proinflammatory cytokine production in S1-stimulated splenocytes. Splenocytes from immunized mice were stimulated with SARS-CoV-2 S1 protein (5 μ*g*/*ml*) for 5 days. The cytokine production of **(A)** TNFα, **(B)** IFNγ, **(C)** IL-12p40, **(D)** IFNβ, **(E)** IL-6, and **(F)** IL-2 from splenocytes was detected by ELISA. Significance differences (*P < 0.05, **P < 0.01, ***P < 0.001) among the different groups are shown in the related figures, and the data are presented as the means ± s.e.m. of mice (*n* = 7).

We further characterized the induction of systemic cytokines in response to the SARS-CoV-2 S1 antigen. Splenocytes of vaccinated mice were stimulated with SARS-CoV-2 S1 protein (5 μ*g*/*ml*), and cytokine secretion in cell culture supernatants was detected at 1, 3, and 5 days post stimulation. Our ELISA data showed that inflammatory cytokines (TNFα, IFNβ, IL-12p40) and Th1-type cytokines (TNFα, IFNγ, and IL-2) were significantly increased in the W4P-RBD group (Figure 6). In particular, W4P-RBD led to significantly higher secretion of IFNγ and IL-12p40 than W-RBD under S1 stimulation (Figures 6B,C). These data suggested that W4P-RBD led to an enhanced cellular immune response skewed to the Th1 response when potently exposed to the SARS-CoV-2 S1 antigen.

## Discussion

Currently, several SARS-CoV-2 vaccine candidates are being developed, among which multiple vaccines target RBDs (18, 29). There is a concern that immune responses elicited by the SARS-CoV-2 vaccine could cause a disease-promoting effect, such as antibody-mediated antibody-dependent enhancement (ADE) or T helper 2 (Th2)-type mediated immunopathology (30). For example, studies on feline coronaviruses have shown that non-neutralizing coronavirus antibodies may cause ADE in feline infectious peritonitis (31). Meanwhile, T helper 2 (Th2)-type immunity induced by SARS-CoV nucleocapsid (N) protein could sometimes cause lung tissue immunopathology due to eosinophil infiltration (32). However, recent studies have demonstrated that SARS-CoV-2 RBD vaccination elicited potent neutralizing responses in preclinical settings without inducing ADE, further supporting the feasibility of RBD-based vaccines as safe SARS-CoV-2 vaccines for clinical use (33).

Here, we introduced a novel RBD targeting the SARS-CoV-2 DNA vaccine, W4P-RBD, with the N-terminal addition of a 33-bp (11 aa) preS1 sequence of the HBV W4P variant that acts as a vaccine adjuvant and characterized its immunogenicity in vaccinated mice compared with the wild-type RBD vaccine (W-RBD) or control. Notably, the *in vitro* transfection of W4P-RBD into multiple cell lines, Vero E6, Huh7, and 293T cells, induced enhanced production of the inflammatory cytokines IL-6 and TNFα compared with that in W-RBD- or mock-transfected cells, suggesting that the added N-terminal HBV preS1 sequences in W4P-RBD acts as an adjuvant to RBD-based DNA vaccines (Figure 1C, Supplementary Figure 1). This *in vivo* vaccine study showing enhanced humoral and cellular immune responses of W4P-RBD strongly supported this notion (Figures 3, 5). Additionally, the *in vivo* immunogenicity study revealed that W4P-RBD immunizations induced remarkably higher levels of SARS-CoV-2 RBD- or S1-specific IgG or IgA antibodies in the serum of vaccinated mice that not only efficiently neutralized pseudotyped and wild-type viruses but also exerted strong antiviral effects by blocking cell entry in an infection model of live virus (Figures 2, 3). Importantly, we further demonstrated that anti-SARS-CoV-2 binding antibodies showing enhanced neutralizing and antiviral activity against infections of pseudotyped and wild-type viruses were induced in the BAL fluid of lungs in W4P-RBD-vaccinated mice, with the potential to prevent lower respiratory disease, which is associated with severe cases of COVID-19 (Supplementary Figures 3B, 4).

In addition to humoral responses, cellular immune responses have been reported to play a pivotal role in controlling coronavirus infections (34). Accumulating evidence suggests that asymptomatic patients or individuals with mild disease typically develop robust T-cell responses (35, 36). Here, we demonstrated that activated IFN-γ- or TNF-producing T-cell responses against SARS-CoV-2 were induced in W4P-RBD-vaccinated mice (Figure 5A). Given that CD4+ T cells could indirectly modulate virus infection by orchestrating antibody production, enhanced T-cell-mediated immune responses, as shown in W4P-RBD vaccination, can contribute to the production and maintenance of IgG and IgA antibodies with potent neutralizing activity (Figure 5B).

Vaccine-induced immunopathology, a potential concern for SARS-CoV-2 vaccines, seems vaccine platform dependent and correlated with low neutralizing antibody titers and a Th2 skewed immune response (37, 38). To date, Th2-mediated immune pathogenesis has not been reported for MERS, SARS-CoV, or SARS-CoV-2 DNA vaccines in mice or non-human primate models (38). Furthermore, W4P-RBD DNA vaccination inducing a dominant Th1 response combined with high titers of nAb in vaccinated mice could reduce the theoretical risk of vaccine-associated enhanced disease (Figures 3A,B, 6A–F). Our data showed no antibody-dependent enhancement (ADE) of SARS-CoV-2 in our *in vitro* studies of W4P-RBD-vaccinated mice (Supplementary Figure 5C). However, this finding must be further substantiated in future SARS-CoV-2 infection experiments.

The present study has some issues to be addressed. First, we did not evaluate role of W4P mutation of W4P-RBD in the induction of increased immune response by using wild type pre-S1/RBD DNA construct as a control. However, we have already checked that preS1 sequences with a W4P mutation, but not wild type, could induce adjuvant effect in HIV-1 p24 DNA vaccine administration (data not shown), demonstrating the role of W4P mutation in induction of increased immune response in DNA vaccine construct. In addition, we have reported that HBV LHBs with W4P mutation can contribute to hepatocellular carcinoma (27) and raise the issue of whether W4P mutation could cause side effect during vaccine application. However, we do not think that combination of 33-bp preS1 sequences with W4P mutation and another vaccine target DNAs different from HBV LHBs could cause some side effects rather than induce adjuvant effect during DNA vaccine application. Meanwhile, we did not find any side effect in W4P-RBD immunized mice. However, the protection efficacy of W4P-RBD vaccine should be evaluated in suitable animal model for COVID-19 in further study. Furthermore, since DNA vaccines encoding RBD alone may have low immunogenicity by the relatively small length of antigen, DNA vaccine with W4P PreS1 region encoding entire spike protein could induce better protection against SARS-CoV-2 infection than W4P-RBD DNA vaccine. It should also be addressed in the further study.

**Figure 7:**
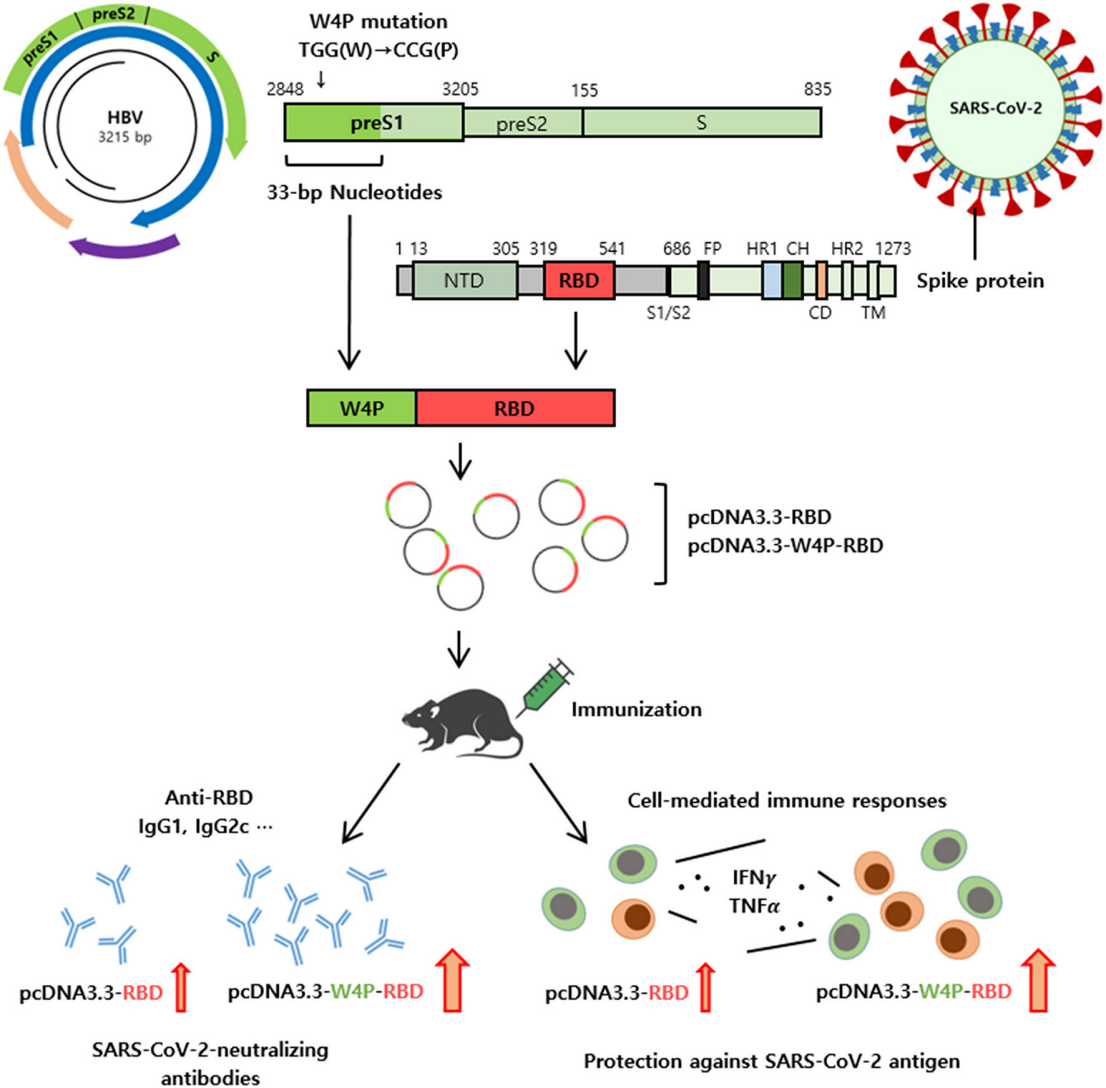
Schematic representation of W4P-RBD as a vaccine candidate against SARS-CoV-2. A novel platform of N-terminal addition of HBV W4P preS1 33-bp sequences for a DNA vaccine against SARS-CoV-2 were developed. The W4P-RBD led to enhanced both humoral and cell-mediated immune response against SARS-CoV-2 in vaccinated mice, demonstrating its feasibility as a DNA vaccine to protect against SARS-CoV-2.

In summary, the W4P-RBD introduced in this study led to an enhanced robust humoral and cell-mediated immune response against SARS-CoV-2 in vaccinated mice, highlighting its feasibility as a novel DNA vaccine to protect against SARS-CoV-2 infection. Additionally, we provide a novel platform (N-terminal addition of HBV W4P preS1 33-bp sequences) for a DNA vaccine development approach capable of strengthening both strong humoral and cellular immune responses in vaccinated mice (Figure 7). Therefore, we expect that this platform can also extend into DNA vaccine development targeting other proteins of SARS-CoV-2, including S1 or S, or to protect against other similar pathogens.

## Materials and Methods

### Design and Synthesis of pcDNA3.3-RBD and pcDNA3.3-W4P-RBD

pcDNA3.3-RBD and pcDNA3.3-W4P-RBD were constructed and synthesized as follows. Briefly, genes encoding RBD (residue 319-541) of the SARS-CoV-2 spike protein were amplified using RBD primers and codon-optimized SARS-CoV-2 Spike ORF mammalian expression plasmid (VG40589; Sino Biological, CN) as the template. Genes encoding W4P-RBD were amplified using primers. Genes encoding the W4P region comprised 33 bp from the first site of the preS1 region, in which the fourth site was found to be a W4P substitution in the HBV genome. All the primer sets and sequences of the W4P region (33 bp) are shown in Supplementary Table 1. The amplified RBD and W4P-RBD genes were inserted into pcDNA3.3 (Invitrogen, USA) using TA cloning according to the manufacturer's instructions. Thereafter, each plasmid DNA was transformed into DH5α *E. coli* cells. DH5α *E. coli* cells with pcDNA3.3-RBD or pcDNA3.3-W4P-RBD were cultured overnight in a 37*C* shaking bed on LB/amp medium. Each plasmid DNA was purified by cesium chloride/ethidium bromide gradient sedimentation.

### Mice Experiments

Specific pathogen-free (SPF) 6–8-week-old male C57BL/6 mice were purchased from Orient Bio. The study was performed according to the guidelines approved by the Institutional Animal Care and Use Committee of Seoul National University (Approval No. SNU 200210-2-1). The mice were vaccinated via intramuscular injection with pcDNA3.3-RBD, pcDNA3.3-W4P-RBD (50 μ*g*/100 μ*l* of PBS), or empty pcDNA3.3 (mock) three times at 1-week intervals. Five weeks after the last vaccination, serum was collected to detect the antibody response and neutralizing antibodies, and the lung, spleen, and inguinal lymph nodes were collected to detect T-cell responses.

### Cell Culture

The monkey kidney cell line Vero E6, human kidney cell line 293T, and human lung cancer cell line Calu-3 were grown in complete Dulbecco's modified Eagle medium (DMEM; Life Technologies, USA) containing 10% FBS and 100 U/ml of penicillin/streptomycin in a humid environment containing CO_2_ and air at 37*C*. The human hepatocarcinoma cell line Huh7 was grown in complete RPMI 1640 supplemented with 10% FBS and 100 U/ml of penicillin/streptomycin. Splenocytes isolated from vaccinated mice were incubated in complete RPMI 1640 supplemented with murine IL-2 for 6 days.

### ELISA

ELISA plates (Corning, USA) were coated with 3 μ*g*/*ml* of SARS-CoV-2 S1 and RBD protein in PBS overnight at 4*C* and blocked in 5% skim milk in PBS. Mouse serum was diluted and added to each well. Following incubation with HRP-conjugated anti-mouse IgG, IgG1, IgG2c, and IgA antibodies, the plates were developed with 3,3′, 5,5′-tetramethylbenzidine. The reactions were stopped with 1 N hydrochloric acid, and the absorbance was measured at 450 nm using a microplate reader (Tecan, CH). The endpoint titer was measured as the highest reciprocal dilution of plasma to indicate 3-fold of the background values.

### Live SARS-CoV-2 Neutralization Assay

DNA vaccine-induced neutralizing antibodies against live SARS-CoV-2 infection were detected using the plaque assay. Mouse serum was diluted and mixed with the same volume of SARS-CoV-2 (150 pfu) and incubated at 37*C* for 2 h. Thereafter, 200 μ*l* of the virus-serum mixtures were transferred to pre-plated Vero E6 cells (5 × 10^5^cells/well) in 24-well-plates. Inoculated cells were incubated at 37*C* for 3 days. For plaque assay, Vero E6 cells were fixed with 4% paraformaldehyde and permeabilized with methanol. The cells were incubated sequentially with primary antibodies against the SARS-CoV-2 nucleocapsid overnight at 4*C*, alkaline phosphatase (AP)-conjugated secondary antibody, and NBT/BCIP. The neutralizing antibody titer (PRNT_50_) was calculated as the highest dilution of serum capable of preventing SARS-CoV-2-induced plaque formation in 50% of that in the positive control.

### Pseudovirus Neutralization Assay

SARS-CoV-2 pseudovirus preparation and neutralization assays were performed to detect DNA vaccine-induced neutralizing antibodies against SARS-CoV-2 pseudovirus infection. Briefly, the plasmids of pNL4-3.luc.R-E- (3418; NIH-AIDS) and pCAGGS encoding SARS-CoV-2 spike glycoprotein S (NR-52310; BEI Resources) were co-transfected into 293T cells. Seventy-two hours after co-transfection, the harvested supernatant media was mixed with PEG-it virus precipitation solution (LV810A-1-SBI) and centrifuged at 1,500 g for 30 min to obtain a lentivirus pellet. Single-use aliquots were stored at −80*C*. The TCID_50_ was determined by infection in Huh7, Calu-3, and Vero E6 cells (39). To evaluate the pseudovirus neutralization activity of mouse serum, an equal volume of ~120 TCID_50_ of pseudoviruses was incubated with serially diluted mouse serum for 2 h at 37*C*, added to Huh7, Calu-3, and Vero E6 cells, and then cultured at 37*C* for 48 h. The cells were lysed using cell lysis buffer (Promega, USA) and transferred into luminometer plates. Luciferase substrate (Promega, USA) was added to the lysates, and the relative luciferase activity was measured using a luminometer (Tecan).

### ICS and Flow Cytometry

Mouse lung, spleen, and inguinal lymph nodes were mashed using a cell strainer and added to the plate (1 × 10^6^/well). For intracellular cytokine staining of IFNγ and TNFα, single cells were then incubated with PMA (50 ng/ml) and ionomycin (1 μ*g*/*ml*), and cytokine release was prevented by treatment with brefeldin A. The cells were then incubated with mAbs, including anti-CD8, anti-CD4, anti-CD3, anti-CD44, and anti-CD62L antibodies, for 30 min on ice. Following fixation/permeabilization, the cells were stained with mAbs, including IFNγ and TNFα. All the antibodies were purchased from BD Biosciences. Fluorescence was measured using FACs LSRII (BD Biosciences, USA) and FlowJo (BD Biosciences, USA) software.

### Western Blotting

Cells were lysed in a radioimmunoprecipitation assay (RIPA; Thermo Fisher Scientific, USA) buffer containing protease inhibitor cocktail and phosphatase inhibitor. Briefly, proteins were separated by 10% Tris-glycine SDS-PAGE and transferred to nitrocellulose membranes. Next, the membranes were blocked in 2% BSA at room temperature. The primary antibodies against GAPDH (Cell Signaling Tech., USA), SARS-CoV-2 nucleocapsid, and SARS-CoV-2 spike RBD (40591-T62; Sino Biological, CN) were incubated with the membrane overnight at 4*C*. After repeated washing, the membranes were incubated with corresponding horseradish peroxidase-conjugated secondary antibodies. The protein blots were examined using ECL reagents.

### IFN Luciferase Reporter Assay

Cell culture supernatants from S1-treated splenocytes were incubated for 4 h with L929 IFN reporter cells containing the ISRE-luciferase construct. The reporter cells were lysed using passive lysis buffer (Promega, USA), mixed with firefly luciferin substrate (Promega, USA), and measured using an illuminometer (Beckman, USA).

### RNA Extraction and qRT-PCR

Total RNA was extracted from pcDNA3.3-transfected and SARS-CoV-2-infected cells using TRIzol reagent (Invitrogen). The expression level of the target gene was analyzed by quantitative reverse transcription-PCR using the ABI 7500 system (Applied Biosystems, USA) and specific primers for IL-6, TNFα, GAPDH, and RNA-dependent RNA polymerase (RdRp), and the SYBR green PCR master mix (Applied Biosystems, USA). All the primer sets are shown in Supplementary Table 2.

### Confocal Microscopy

SARS-CoV-2-infected cells were fixed and inactivated with 4% paraformaldehyde for 1 h following UV irradiation. The cells were permeabilized with 0.1% Triton X-100 for 15 min and blocked with 1% BSA for 1 h at room temperature. Thereafter, the cells were labeled with rabbit anti-nucleocapsid antibody (diluted 1:500), incubated overnight at 4°C and labeled with goat anti-rabbit IgG conjugated with Alexa Fluor 488 (diluted 1:1,000) for 1 h at room temperature. Nuclei were stained with DAPI (Invitrogen), and the cells were visualized by fluorescence microscopy using a confocal laser scanning microscope system (Olympus FV3000).

### Statistical Analysis

Values are shown as the mean ± standard error (s.e.m.) and were analyzed using GraphPad Prism 5.01 statistical software (GraphPad, USA). The significance differences among multiple groups were analyzed by one-way analysis of variance (ANOVA) followed by Dunnett's multiple comparison test. Significance differences (^*^P < 0.05, ^**^P < 0.01, ^***^P < 0.001) among the different groups are shown in the related figures. Pearson's correlations were calculated to define correlations throughout the manuscript.

## Data Availability Statement

The original contributions presented in the study are included in the article/Supplementary Material, further inquiries can be directed to the corresponding author/s.

## Ethics Statement

The animal study was reviewed and approved by Institutional Animal Care and Use Committee (IACUC) of Seoul National University College of Medicine (SNU 200210-2-1).

## Author Contributions

HJ and B-JK designed the research. HJ performed and analyzed overall experiments and wrote the manuscript. Y-MC supported animal experiments. HS supported DNA work. B-JK supervised overall experiments and wrote the manuscript. All authors contributed to the article and approved the submitted version.

## Conflict of Interest

The authors declare that the research was conducted in the absence of any commercial or financial relationships that could be construed as a potential conflict of interest.
