# Supplementary material for: A Novel DNA Vaccine Against SARS-CoV-2 Encoding a Chimeric Protein of Its Receptor-Binding Domain (RBD) Fused to the Amino-Terminal Region of Hepatitis B Virus preS1 With a W4P Mutation
Source: Front Immunol. 2021 Feb 26;12:637654. doi: 10.3389/fimmu.2021.637654 (PMC7959807; doi:10.3389/fimmu.2021.637654)
Supplement: Supplementary file 1 [file Data_Sheet_1.pdf]

Target region	Primer name	Sequence
**RBD of SARS-CoV-2 (residue 319-541)**	RBD_F	5'- ATG AGG GTC CAA CCA AC -3'
	RBD_R	5'- TCA GAA GTT CAC ACA CTT G -3'
**W4P-RBD**	W4P-RBD_F	5'- ATG GGA GGT CCG TCT TCC AAA CCT CGA CAA GGC AGG GTC CAA CCA ACA G -3' (W4P sequence)
	RBD_R	5'- TCA GAA GTT CAC ACA CTT G -3'

Supplementary table 1. Primers used in synthesis of pcDNA3.3-RBD and pcDNA3.3-W4P-RBD

Primer name	Sequence
IL6_F	5’- ACAGCCACTCACCTCTTCAG -3’
IL6_R	5’- CCATCTTTTTCAGCCATCTTT -3’
TNFα_F	5’- CCCGAGTGACAAGCCTGTAG -3’
TNFα_R	5’- GATGGCAGAGAGGAGGTTGAC -3’
GAPDH_f	5′- GGATTTGGTCGTATTGGG -3′
GAPDH_R	5′- GGAAGATGGTGATGGGATT -3′
RdRp_F	5′-GTGARATGGTCATGTGTGGCGG-3′
RdRp_R	5′-CARATGTTAAASACACTATTAGCATA-3′

Supplementary table 2. Primers used in RT-qPCR

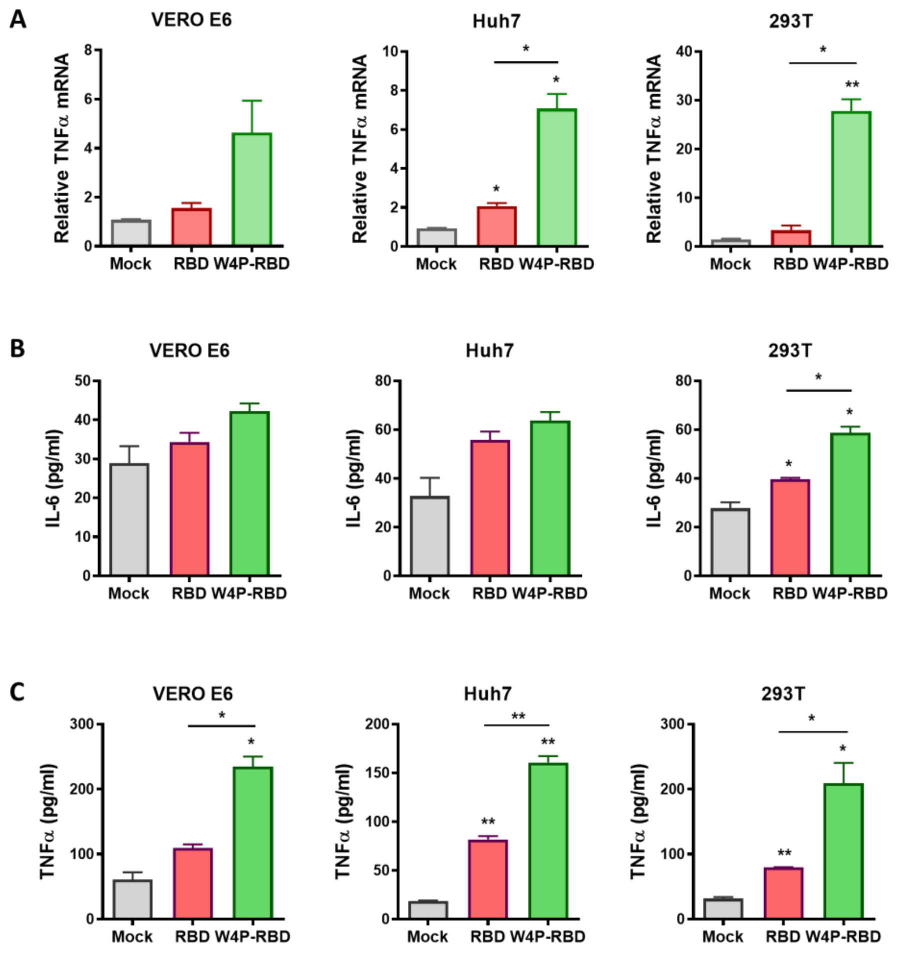

Supplementary figure 1. Cytokine induction in Vero E6, Huh7, and 293T cells transfected with pcDNA3.3-RBD, pcDNA3.3-W4P-RBD, or mock.

Vero E6, Huh7, and 293T cells seeded in 6-well plates (1×10^6 cells/well) were transfected with 2.5 μg of W-RBD, W4P-RBD, or mock. 48 hours post-transfection, cytokines (**B**) IL-6 and (**C**) TNFα in the culture supernatant were detected by ELISA. The cells were harvested, and the mRNA expression levels of (**A**) TNFα were detected by qRT-PCR. Significance differences ($*P < 0.05$, $**P < 0.01$, $***P < 0.001$) among the different groups are shown in the related figures, and the RNA and ELISA data are presented as the means $\pm$ s.e.m. of three independent experiments.

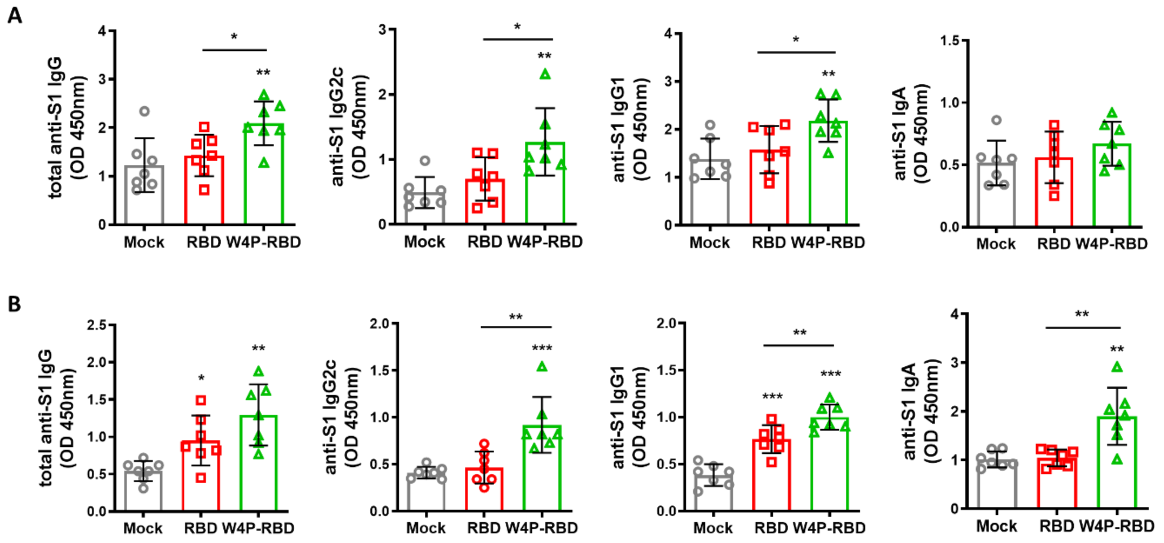

Supplementary figure 2. SARS-CoV-2 S1-specific IgG and IgA production in the (A) serum and (B) BAL of mice immunized with W-RBD, W4P-RBD, or mock.

SARS-CoV-2 S1-specific total IgG, IgG1, IgG2c, and IgA antibodies in the serum and BAL of C57BL/6 mice immunized with W-RBD, W4P-RBD, or mock three times at one-week intervals were detected by ELISA. Significance differences (*$P < 0.05$, **$P < 0.01$, ***$P < 0.001$) among the different groups are shown in the related figures, and the data are presented as the means ± s.e.m. of mice (n=7).

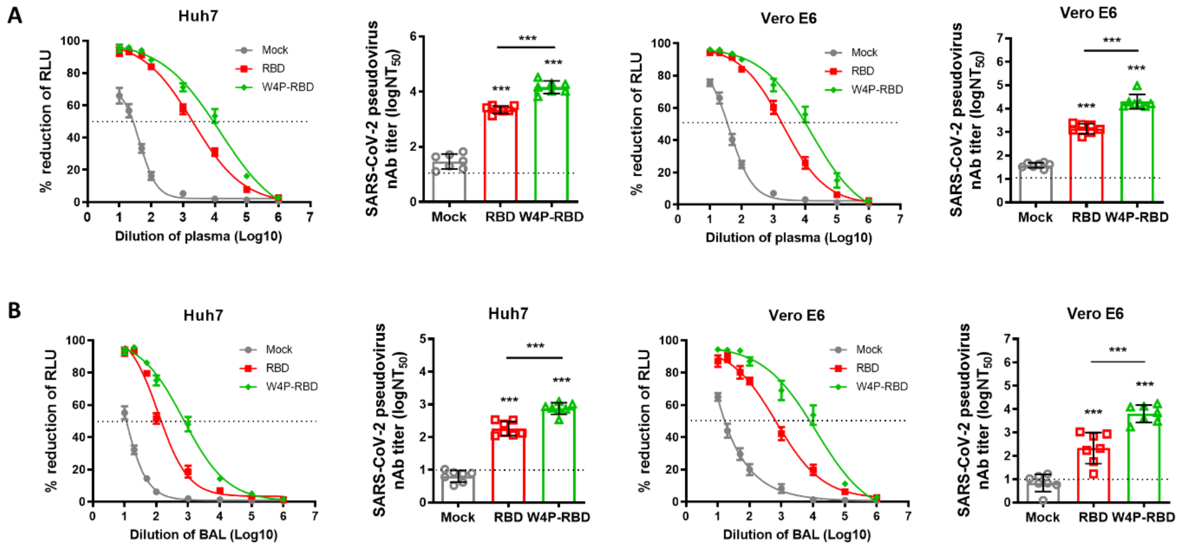

Supplementary figure 3. SARS-CoV-2 pseudovirus neutralization efficacy of the serum and BAL fluid of mice immunized with W-RBD, W4P-RBD, or mock.

SARS-CoV-2 pseudoviruses were collected by cotransfection of the pNL4-3.luc plasmids. RE and pCAGGS encode SARS-CoV-2 spike glycoprotein into 293T cells. Pseudovirus infection efficacy in Huh7 and Vero E6 cells was calculated by the luciferase assay using cell lysates infected with SARS-CoV-2 pseudovirus. The (**A**) Serum and (**B**) BAL fluid of mice immunized with W-RBD, W4P-RBD, or mock were diluted and incubated with an equal volume of ~120 $TCID_{50}$ of pseudovirus added to Huh7 and Vero E6 cells. Two days post-infection, the relative luciferase units were calculated by the luciferase assay using cell lysates. Significance differences (*$P < 0.05$, **$P < 0.01$, ***$P < 0.001$) among the different groups are shown in the related figures, and the data are presented as the means $\pm$ s.e.m. of mice (n=7).

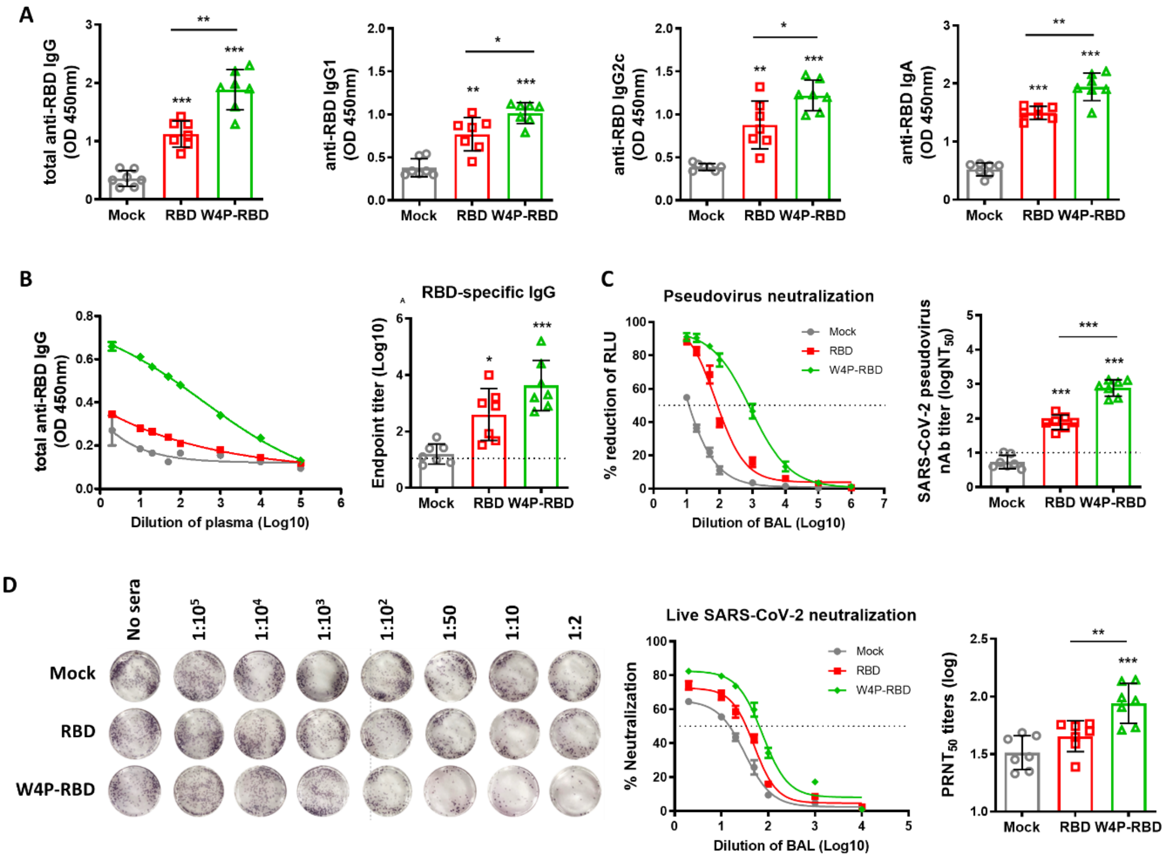

Supplementary figure 4. Neutralizing activity against SARS-CoV-2 in BAL fluid from mice immunized with W-RBD, W4P-RBD, or mock.

C57BL/6 mice were immunized with W-RBD, W4P-RBD (50 µg/mouse), or mock three times at one-week intervals. BAL fluid was collected at five weeks post-immunization to detect antibody responses against SARS-CoV-2. (**A**) Subtype antibody responses in BAL specific to SARS-CoV-2 RBD proteins were detected by ELISA. (**B**) BAL fluid was assessed with different dilution factors for IgG against the SARS-CoV-2 RBD protein using ELISA. (**C, D**) The 50% neutralizing antibody titer (NT_{50}) and 50% plaque reduction neutralizing antibody ($PRNT_{50}$) titer against live SARS-CoV-2 were calculated using the neutralization assay for SARS-CoV-2 pseudovirus and live SARS-CoV-2, respectively. Significance differences (**P* $<$ 0.05, ***P* $<$ 0.01, ****P* $<$ 0.001) among the different groups are shown in the related figures, and the data are presented as the means $\pm$ s.e.m. of mice (n=7).

A

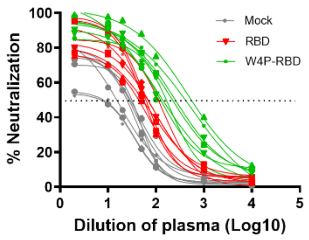

PRNT$_{50}$ plasma dilution	Mock	RBD	W4P-RBD
1	1:38	1:94	1:561
2	1:39	1:60	1:200
3	1:36	1:76	1:112
4	1:18	1:132	1:204
5	1:35	1:54	1:462
6	1:48	1:77	1:122
7	1:42	1:66	1:254

B

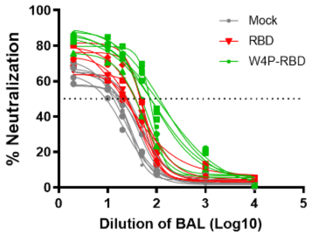

PRNT$_{50}$ BAL dilution	Mock	RBD	W4P-RBD
1	1:28	1:58	1:54
2	1:42	1:57	1:98
3	1:29	1:55	1:81
4	1:21	1:45	1:221
5	1:22	1:39	1:95
6	1:48	1:50	1:136
7	1:45	1:25	1:59

C

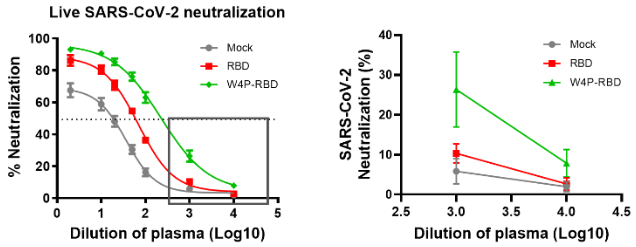

Supplementary figure 5. Live SARS-CoV-2 plaque reduction efficacy of (A) serum and (B) BAL fluid from mice immunized with W-RBD, W4P-RBD, or mock.

50% of live SARS-CoV-2 neutralizing antibody (PRNT$_{50}$) titer was calculated against live SARS-CoV-2 infection in Vero E6 cells. Serum and BAL fluid were collected from mice immunized with W-RBD, W4P-RBD, or mock and diluted, followed by incubation with an equal volume of ~150 pfu of live SARS-CoV-2. 72 hours post-infection, the PRNT$_{50}$ titer was calculated using the plaque assay using the infected cells. (**C**) Antibody-dependent enhancement (ADE) via live SARS-CoV-2 neutralization of serum from immunized mice with W-RBD, W4P-RBD, or mock. Significance differences (*$P < 0.05$, **$P < 0.01$, ***$P < 0.001$) among different groups are shown in the related figures, and the data are presented as the means ± s.e.m. of mice (n=7).

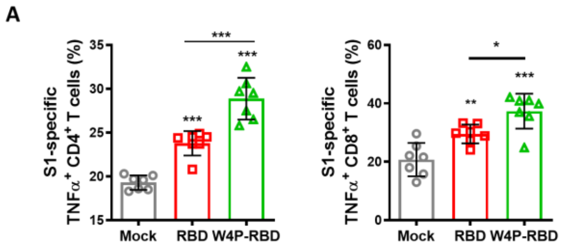

Supplementary figure 6. Induction of (A) SARS-CoV-2 S1-specific T cells in splenocytes from mice immunized with W-RBD, W4P-RBD, or mock.

Significance differences (*P < 0.05, **P < 0.01, ***P < 0.001) among the different groups are shown in the related figures, and the data are presented as the means ± s.e.m. of mice (n=7).
